# Elucidating the Conducting Mechanisms in a Flexible Piezoresistive Pressure Sensor Using Reduced Graphene Oxide Film in Silicone Elastomer

**DOI:** 10.3390/s23052443

**Published:** 2023-02-22

**Authors:** Golezar Gilanizadehdizaj, Debes Bhattacharyya, Jonathan Stringer, Kean Aw

**Affiliations:** Department of Mechanical and Mechatronics Engineering, The University of Auckland, Auckland 1010, New Zealand

**Keywords:** flexible piezoresistive sensor, Schottky emission, thermionic emission, Ohmic conduction

## Abstract

Sensors as a composite film made from reduced graphene oxide (rGO) structures filled with a silicone elastomer are soft and flexible, making them suitable for wearable applications. The sensors exhibit three distinct conducting regions, denoting different conducting mechanisms when pressure is applied. This article aims to elucidate the conduction mechanisms in these sensors made from this composite film. It was deduced that the conducting mechanisms are dominated by Schottky/thermionic emission and Ohmic conduction.

## 1. Introduction

Electronic skins have attracted significant interest due to their broad applications in our daily lives. Flexible pressure sensors form one of the essential parts of any electronic skin. Achieving high-performance electronic skins requires making lightweight pressure sensor arrays with high sensitivity, flexibility, and adaptability. Nowadays, there is a growing demand for flexible pressure sensors in several applications, such as healthcare monitoring [1,2], wearable electronics [3,4], electronic skin [5,6], human–machine interface [7,8], etc. Most sensors use metallic foils and rigid semiconductors, which have shown several limitations, such as low sensitivity and low flexibility, usually less than 5%, making it challenging to apply them in applications requiring flexible contact or wearable devices [9,10]. Capacitive pressure sensors show good stability, high response speed, low power consumption, and a straightforward and inexpensive production technique. However, they are limited by sensitivity [11,12] and have an inherent limitation of low sensitivity at large pressures. Capacitive pressure sensors are also sensitive to external noise sources from electromagnetic waves and show high hysteresis due to the viscoelastic nature of rubber dielectrics [13,14]. Piezoelectric sensors are self-powered and have high sensitivity and fast response times to applied dynamic pressure; however, piezoelectric-induced pressure sensors present challenges in measuring the static signal generated by the transient flow of electrons in an external load driven by a piezoelectric potential generated by dynamic stress. They have a restricted selection of sensing materials, which are electrically polarized compared to other sensors. The manufacturing process is also expensive and complicated [15,16,17]. Flexible triboelectric pressure sensors with high output voltage and low energy consumption can be quickly and affordably made. However, the low environmental tolerance of these sorts of sensors significantly negatively impacts the triboelectric pressure sensor’s performance [18,19,20]. Humidity and temperature fluctuation results in the loss of surface charge, impairs output performance, and creates stability problems. Additionally, consistency issues are brought on by the triboelectric surface’s unequal initial charge. Furthermore, a triboelectric pressure sensor that can exhibit an ultrawide linearity range is rarely reported [21,22,23,24], and often shows a relatively low sensitivity due to the limited contact areas. Therefore, developing cost-effective, flexible pressure sensors with a simple strategy with sufficient sensitivity, wide operating pressure range, and environmental durability remains challenging. An alternative could be piezoresistive pressure sensors. Piezoresistive pressure sensors are among the most preferred flexible sensors due to their simple structure, low cost, quick processing, and straightforward fabrication [25,26,27].

The working principle of a piezoresistive flexible pressure sensor is based on the change in pressure applied from the outside, which is reflected as a change in the corresponding resistance value. Having pressure sensors in an array can provide more information to be derived, allowing better decision making for many applications. However, while cost-effective, traditional piezoresistive sensors, based on semiconductors and metal foils, are relatively rigid, limiting their wearable electronics applications [28]. Compared to other materials, carbon nanomaterials such as graphene, carbon black, and carbon nanotubes possess high sensitivity, wide working range, simple and low-cost manufacturing process, superior flexibility, and stability under ambient conditions [29,30,31], and can be integrated to make soft, flexible, and wearable sensors. We have reported [32] on the facile fabrication of flexible piezoresistive pressure sensor arrays with excellent mechanical stability, high sensitivity, low hysteresis, and a wide working range (0–30 kPa) of pressure using a film made from a 3D network of rGO that is encapsulated by Ecoflex 00-30 on a flexible printed circuit board (FPCB). The sensor showed two linear regions and a remarkable sensitivity of 0.13 kPa^−1^ in the pressure range of <10 kPa and 0.05 kPa^−1^ in the range of >10 kPa, covering a broad spectrum of testing conditions. We demonstrated that it could provide a pressure map and intensity, providing tactile information on the pressure exerted on our prototype with a 4 × 4 array [32]. These different regions could indicate the presence of various conducting mechanisms. Evaluating the conduction mechanism is very important since it improves comprehension on how the sensor behaves under different pressures. In this report, experiments were conducted to measure the variation in resistance (*R*) with temperature (*T*) and current (*I*) with voltage (*V*). The *R* versus *T* and *I* versus *V* were plotted and curved fitted with the equations of different conducting mechanisms. As a result, the conducting mechanism can be deduced based on the equation that best fits the plot.

## 2. Results and Discussion

### 2.1. Fabrication Method

**Figure 1:**
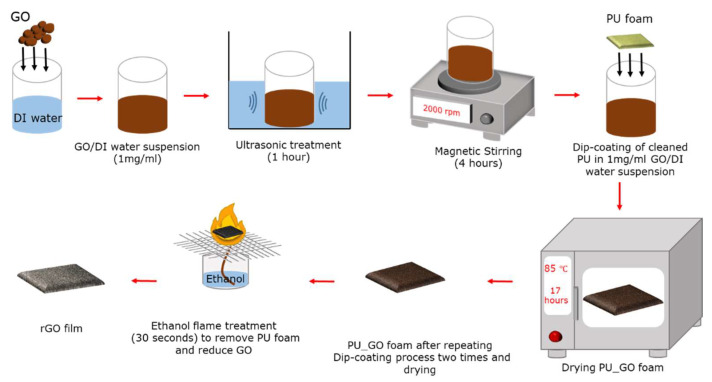
Fabrication process of rGO film.

Figure 1 depicts the fabrication process of rGO film. Graphene oxide (GO) was dispersed in deionized (DI) water with a 1 mg/mL concentration and mixed well using an ultrasonic treatment for an hour, followed by 4 h of magnetic stirring at 2000 rpm to create a GO/DI suspension. A polyurethane (PU) sponge cut into the desired size, 40 mm (length) × 40 mm (width) × 3 mm (thickness), was washed with DI water and ethanol three times and then dried in an oven (85 °C) for 4 h to remove any remaining moisture. The PU sponge was dip-coated into the GO/DI suspension prepared earlier. The PU sponge was squeezed and re-soaked into the GO/DI suspension three times to ensure sufficient GO was absorbed into the sponge. The dip-coating process was repeated twice to ensure an adequate amount of GO was absorbed into the sponge. It was then dried in an oven at 85 °C for 17 h. Finally, the GO-coated PU sponge was exposed to an ethanol flame for 30 s to reduce the GO and burn off the sponge, resulting in a conductive rGO film [32].

**Figure 2:**
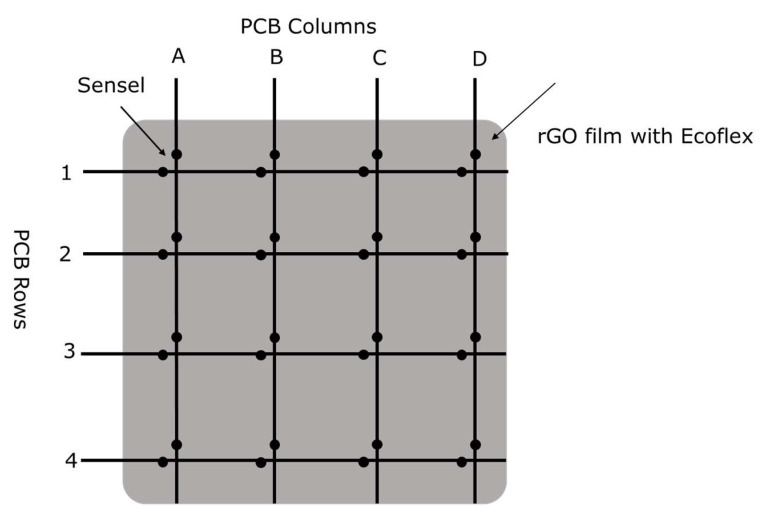
Schematic view of the double-layer FPCB for a 4 × 4 sensor array [32].

**Figure 3:**
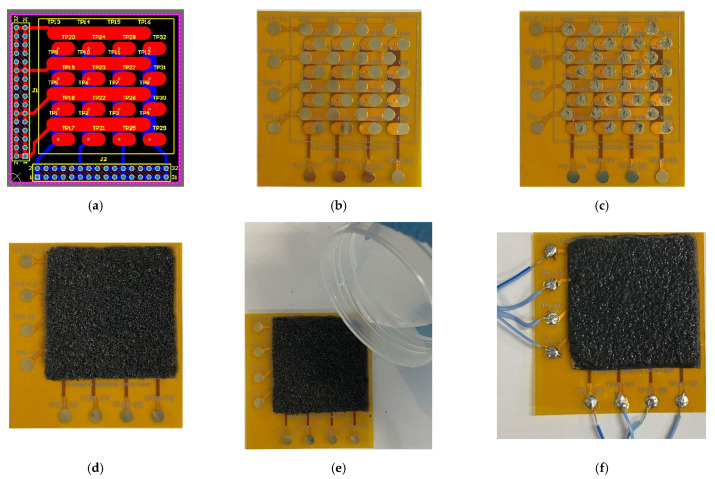
Fabrication process flow of the tactile pressure sensor with an FPCB and rGO film (**a**) double-layer PCB CAD design, (**b**) FPCB for 4 × 4 sensels, (**c**) FPCB with conductive silver grease applied, (**d**) rGO film attached to FPCB, (**e**) pouring of Ecoflex 00-30 onto the rGO film, (**f**) a completed tactile sensor [32].

A double-layer FPCB with electrodes was used to make a sensel array of 4 × 4 with the rGO film. Each sensel can be referenced with its <column><row>, such as sensel A1, A2, …, D4, etc. (Figure 2). RGO film was attached to the FPCB with conductive silver grease. Two parts of Ecoflex 00-30 were mixed 1A:1B by weight and placed in a vacuum chamber to eliminate the bubbles. Then, it was poured onto the attached rGO film and cured in the oven at 50 °C for 4 h. The silicone Ecoflex is an elastomeric material holding the rGO film together, making the array flexible and stretchable. Figure 3 shows the steps in creating a tactile pressure sensor using a 4 × 4 sensel array on rGO film and an FPCB [32].

### 2.2. Sensitivity of the rGO-Based Flexible Pressure Sensor

**Figure 4:**
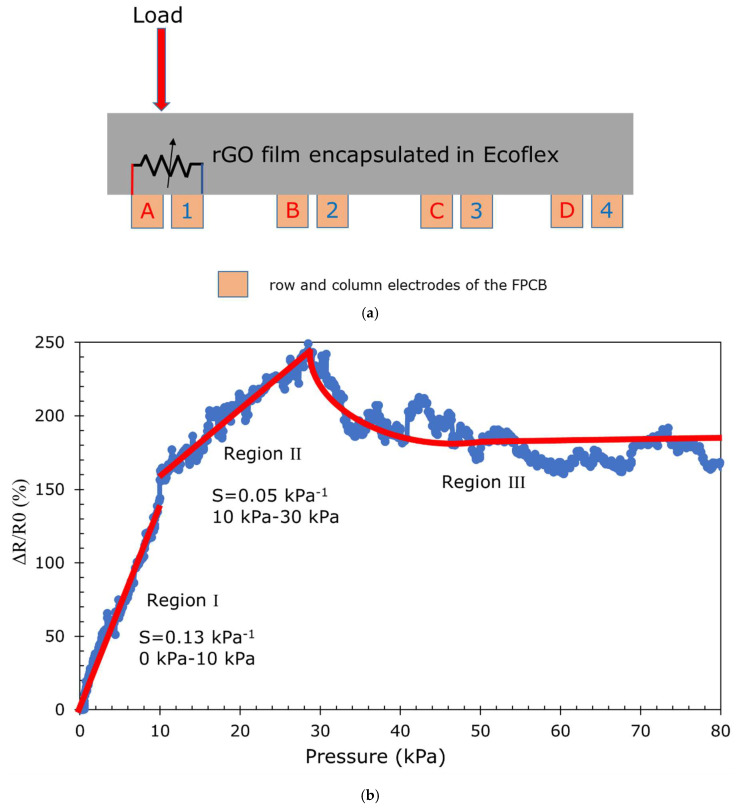
(**a**) A cross-section of the sensor showing four sensels, (**b**) relative resistance change in the sensor with pressure showing three different regions.

Before evaluating the sensitivity, the sensor was preloaded at 30 kPa pressure for 30 min to ensure that all weak bonds were broken and a steady state was achieved. As illustrated in Figure 4a, the resistance was measured via the electrodes pair in the form of the FPCB attached at the bottom side of the rGO film with an LCR meter when a compressive force was applied on sensel A1 with a compression rate of 5 mm/min. The sensitivity of these rGO-based piezoresistive sensors can be calculated with:

(1) $$S=\frac{\left(\frac{△R}{R_{0}}\right)}{P}\;\;\;\;\;\;\;\;\;\;\;\;△R=R-R_{0}$$

where the *R* relates to the resistance under the pressure *P* and the *R*_0_ refers to the initial zero load resistance of the piezoresistive sensor. Figure 4b demonstrates the sensor’s relative resistance at different pressures. The slope of the graph indicates the sensitivity of the sensor.

As illustrated in Figure 4b, the sensor exhibits three distinct regions. There are two linear regions (I and II) with a sensitivity of 0.13 kPa^−1^ at the range of <10 kPa and 0.05 kPa^−1^ at 10–30 kPa. Region I shows that when low pressure (<10 kPa) is applied to the sensor, the resistance increases with a steep gradient up to 10 kPa. In the pressure range of 10 kPa to 30 kPa (Region II), with increasing pressure, the resistance increases but has a lower rate than Region I. As the pressure goes beyond 30 kPa, (Region III), the resistance gradually reduces and then plateaus off. These different regions indicate the presence of various conducting mechanisms, which are described in the following section.

### 2.3. Conducting Mechanisms of the rGO-Based Flexible Pressure Sensor

**Figure 5:**
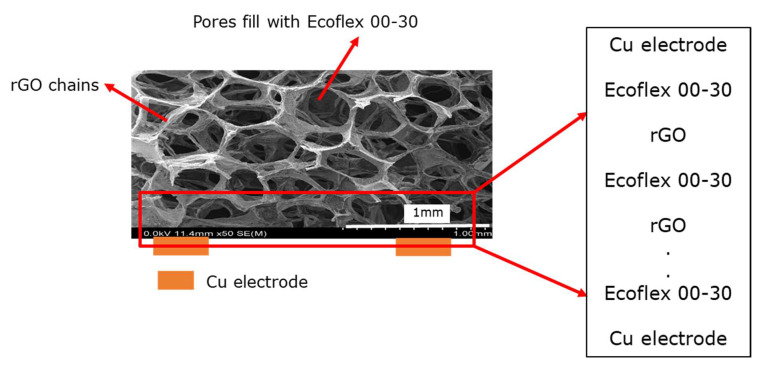
An illustration showing the rGO is placed on top of copper electrodes to form a sensing element.

The pressure sensor is made of a 3D network film of rGO filled with Ecoflex 00-30, and electrodes are attached at the bottom [32]. In other words, the conducting mechanism from one electrode through the sensor to the other electrode can be viewed as electrical conduction from the metal(copper)–insulator(Ecoflex 00-30)–semiconductor(rGO)–insulator(Ecoflex 00-30)–semiconductor (rGO)–………–metal (electrodes), as illustrated in Figure 5. Hence, this is an analogy of the conduction in a metal–insulator–semiconductor (MIS) structure. The dominant conduction mechanisms in the MIS structure can be experimentally deduced. The dielectric film in this sensor is Ecoflex 00-30 and is a silicone-based insulator with a relative permittivity of 2.8 [33]. The main parameters affecting the conduction are the barrier heights of the metal (Cu)-dielectric interface and the effective mass of the conduction carrier [34]. Generally, the mechanisms by which the conduction carriers are transported through the system can broadly be classified as electrode-limited conduction mechanisms and bulk-limited conduction mechanisms [35,36,37]. 

The electrode-limited conduction mechanisms rely on the electrical properties at the electrode–dielectric contact. The barrier height is the most significant factor in this type of conduction mechanism [38]. The electrode-limited conduction mechanisms include (i) Schottky or thermionic emission, (ii) Fowler–Nordheim tunneling, (iii) direct tunneling, and (iv) thermionic field emission [39]. The bulk-limited conduction mechanisms depend on the electrical properties of the dielectric itself. The most critical parameter in this conducting mechanism is the trap energy levels in the dielectric films [34]. The bulk-limited conduction mechanisms include (i) hopping conduction, (ii) Poole–Frenkel emission, (iii) Ohmic conduction, (iv) and ionic conduction [40]. Table 1 shows the classification and equations for various conduction mechanisms in a dielectric film.

**Table 1:** Classification of the conduction mechanisms and their equations.

Conduction Mechanisms	
Electrode-Limited Conduction Mechanism	Bulk-Limited Conduction Mechanism
➢ Schottky or thermionic emission [41] $J=A^{*}T^{2}\;\exp\;\left[\frac{-q(\emptyset_{B}-\sqrt{qE/4\pi \varepsilon_{r}\varepsilon_{0}}}{kT}\right]$ $A^{*}=\frac{4\pi qk^{2}m^{*}}{h^{3}}=\frac{120m^{*}}{m_{0}}$	➢ Poole-Frankel (P-F) emission [42] $J=q\mu N_{C}E\;exp\;\left[\frac{-q\left(\phi_{T}-\sqrt{qE/\pi \varepsilon_{r}\varepsilon_{0}}\right)}{kT}\right]$
➢ Fowler–Nordheim (F–N) tunneling [43] $J=\frac{q^{3}E^{2}}{8\pi hq\emptyset_{B}\;}exp\;\left[\frac{-8\pi {\left(2qm^{*}{}_{t}\right)}^{1/2}}{3hE}\emptyset_{B}{}^{3/2}\right]$	➢ Hopping conduction [44] $J=qan\nu \;exp\left[\frac{qaE}{kT}-\frac{E_{a}}{kT}\right]\;$
➢ Direct tunneling [45] J∼exp −8πqϕB322meff3hqE3V2ϕB	➢ Ohmic conduction [46] J=σE=nqμE $n=N_{C}\;exp\left[\frac{-\left(E_{C}-E_{F}\right)}{kT}\right]\;$
➢ Thermionic field emission [38] $J=\frac{q^{2}\sqrt{m}{\left(kT\right)}^{1/2}E}{8\hbar^{2}\pi^{5/2}}\;exp\;\left(\frac{-q\emptyset_{B}}{kT}\right)\;exp\left[\frac{\hbar^{2}q^{2}E^{3}}{24m{\left(kT\right)}^{3}}\right]\;$	➢ Ionic Conduction [46] $J=J_{0}\;exp\left[-\left(\frac{q\phi_{B}}{kT}-\frac{Eqd_{s}}{2kT}\right)\right]\;$

*J* = current density, $A^{*}$ = effective Richardson constant, *T* = absolute temperature, q = electron charge, $\emptyset_{B}\;$ = barrier height, E = electric field across the dielectric, $\varepsilon_{r}$ = dynamic dielectric constant, $\varepsilon_{0}$ = permittivity in vacuum, *k* = Boltzmann’s constant, *h* = Planck’s constant, m^*^_t_ = tunnelling effective mass in dielectric, *m_eff_* = tunnelling effective mass in dielectric, *V* = voltage across the dielectric, *m* = effective mass in dielectric, ħ = reduced Planck’s constant, μ = electronic drift mobility, *N_C_* = density of states in the conduction band, $q\phi_{T}$ = trap energy level, *a* = mean spacing between trap sites, *n* = electron concentration in the conduction band, ν = frequency of the thermal vibration of electrons at trap sites, *E_a_* = the energy level from the trap sites to the bottom of the conduction band (*E_C_*), σ = electrical conductivity, *E_F_* = Fermi level, *J*_0_ = proportional constant, *d_s_* = spacing of two nearby jumping sites, *d* = thickness of the film.

From the equations of the conduction mechanisms in Table 1, the current density (*J*), which is dependent on the resistance (*R*), is affected by either voltage or temperature. From the equations in Table 1, it is clear that *T* is the most common parameter that affects *R*. Thus, the conductive mechanism can generally be deduced from the *R* versus *T* plot, but the *I* versus *V* plot was also used to distinguish them when there were mechanisms that exhibited similar *R* versus *T* behavior.

#### 2.3.1. Region I: Conduction Mechanism of the Sensor at Low Pressure (0–10 kPa) 

To investigate the conduction mechanism of the sensor in Region Ⅰ, the sensor was placed into an oven without any pressure applied (0 kPa), and its resistance was measured using an LCR meter at different temperatures (0 °C to 80 °C). In addition, the *I* versus *V* was also measured. Then, the *R* versus *T* and *I* versus *V* were plotted and curve-fitted with different conduction mechanisms equations. Curve-fitting was conducted on the plots for the various mechanisms in Table 1 and the R^2^ of the curve-fitting is summarized in Table 2. From the curve-fitting of the *R* versus *T* plots, the Schottky or thermionic conduction seems to fit relatively well.

**Figure 6:**
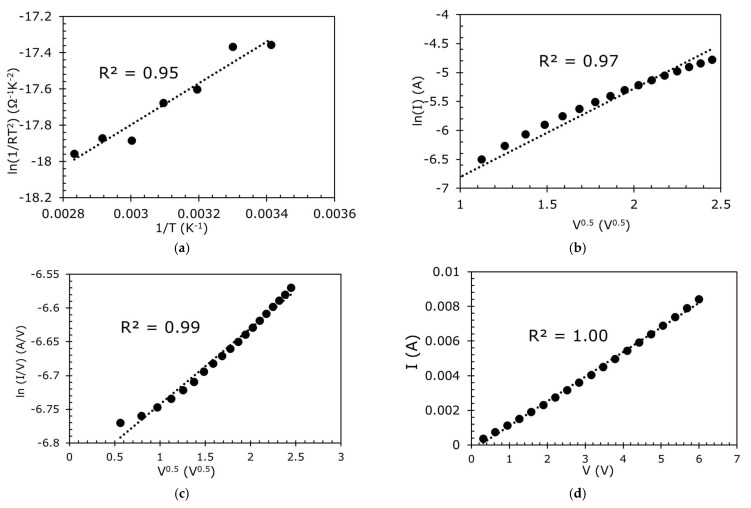
(**a**) Temperature variation in the sensor and its fit to Schottky or thermionic emission, *I*–*V* plots of the sensor and fitted with (**b**) Schottky or thermionic emission, (**c**) Poole–Frenkel, and (**d**) Ohmic conduction in Region I.

To further understand the conduction mechanism, curve-fitting of the *I* versus *V* plots was also performed. Poole–Frenkel and Ohmic conduction, despite not fitting well in the *R* versus *T* plots (See Table 2), have good fitting in the *I* versus *V* plots (Figure 6). It is believed that Schottky/thermionic emission, which is an electrode-limited conduction mechanism, is the dominant conduction mechanism where electrons can overcome the energy barrier at the metal–dielectric interface to travel over the dielectric toward the semiconductor [34], but with the presence of other conduction mechanisms such as Poole–Frenkel and Ohmic conduction. 

**Figure 7:**
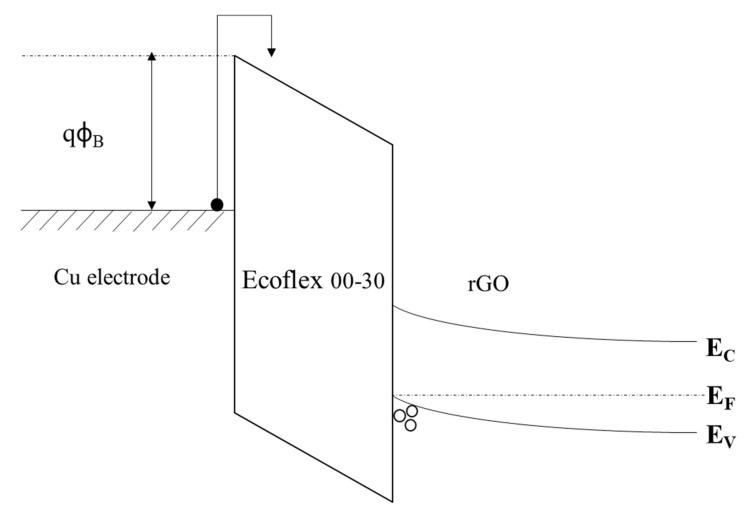
A band diagram showing the Schottky/thermionic emission in Region I.

Regarding the conduction mechanism, it can be hypothesized that the deformation of the sensor under compressive load, with low pressures (0–10 kPa), primarily causes an orthogonal tensile strain and a corresponding increase in the work function and, hence, resistance increase [47]. The work function of the electrodes is important as it determines the barrier height (*qΦ_B_*) in the Schottky/thermionic band–diagram model (Figure 7). In addition, the compression of the 3D rGO chain structure causes them to be squeezed closer together and could have some bulk properties, leading to some bulk-limited conduction mechanisms such as Ohmic and Poole–Frenkel.

**Figure 8:**
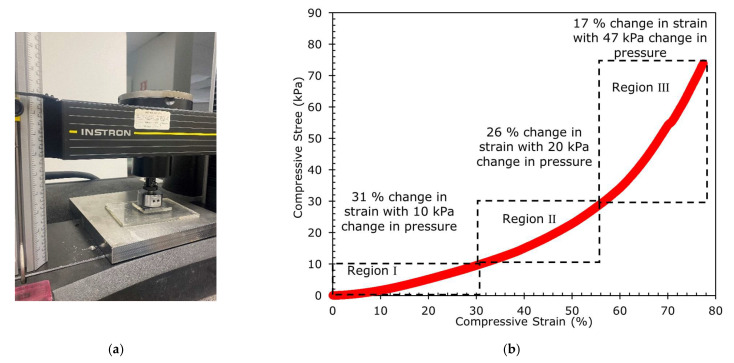
(**a**) Instron machine 5866 used to conduct the compression stress, and the obtained (**b**) compression stress and versus compression strain plot.

To study how the sensor deforms under different loads, three composite films with a length (*l*) of 47 mm, a width (*w*) of 24 mm, and a thickness (*t*) of 3 mm were prepared and characterized with a universal testing machine (Figure 8a). The extension rate (0.01 mm/s) controls the test, which controls the constant compression speed (the cross-head pressure). The compression stress versus compression strain was obtained from Instron and shown in Figure 8b. It is clear that the relationship is non-linear. 

As the composite film is compressed vertically, there is some orthogonal (horizontal) strain, and this causes the barrier height in the Cu-Ecoflex 00-30 (*qΦ_B_*) to increase [47], which increases the resistance of the sensor as the electrons require higher energy to overcome the barrier height. It is believed that this causes the change in resistance in Region I of Figure 4b. Further from Figure 8b, the compression strain increases more rapidly when the pressure increases from 0 to 10 kPa (strain change from 0% to 31% with a pressure change of 10 kPa), which leads to the corresponding orthogonal strain and increases the resistance with pressure in Region I.

#### 2.3.2. Region II: Conduction Mechanism of the Sensor at Medium Pressure (10–30 kPa)

**Figure 9:**
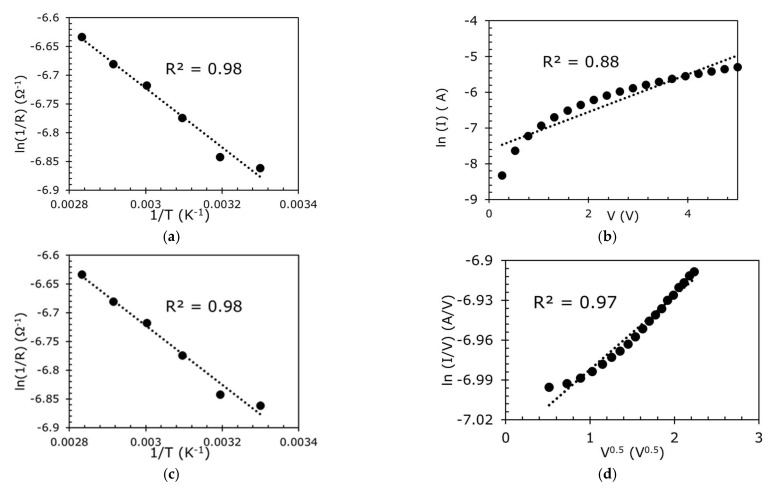

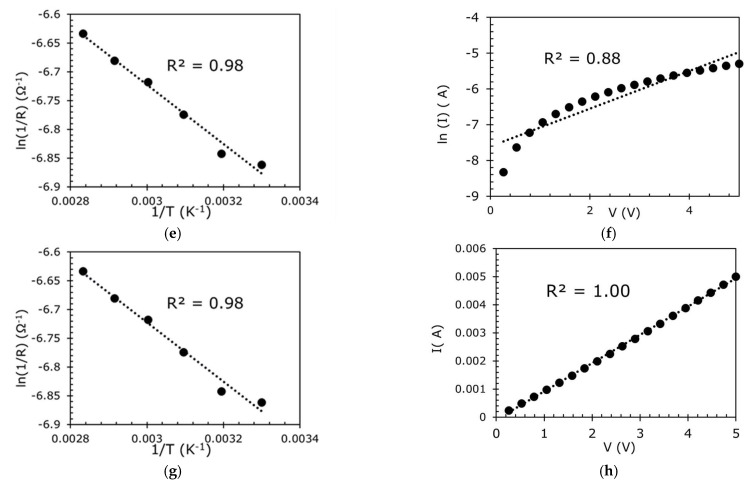
*R* versus *T* and *I* versus *V* curves of the sensor at 28.5 kPa (Region II) and their fit to (**a**,**b**) hopping conduction; (**c**,**d**) Poole–Frankel; (**e**,**f**) ionic conduction; (**g**,**h**), Ohmic conduction.

**Figure 10:**
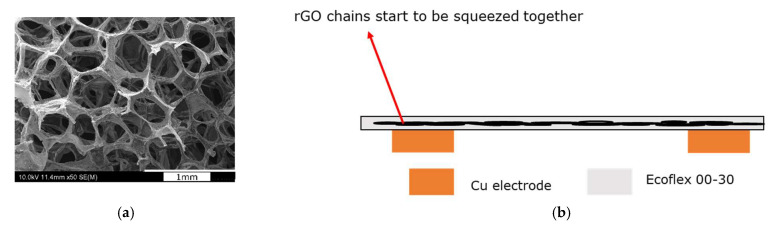
(**a**) Scanning electron microscope (SEM) image of the 3D rGO structure. (**b**) Illustration showing the collapse of the 3D rGO of the sensor under high pressure, acting as quasi-bulk material.

To represent Region II, two clamps applied a pressure of 28.5 kPa to the sensor. It was placed in an oven to measure the sensor’s resistance using an LCR meter at different temperatures. *R* versus *T* curves show a good fit with hopping conduction, Poole–Frankel emission, ionic conduction, and Ohmic conduction with R^2^ > 0.98 (Figure 9a,c,e,g). The *I* versus *V* curves were also plotted for hopping conduction, Poole–Frankel emission, Ohmic, and ionic conduction (Figure 9b,d,f,h). It is clear that the conduction in Region II is dominated by Ohmic conduction, as the *R* versus *T* and *I* versus *V* are extremely well-fitted with the equation related to Ohmic conduction. As the pressure is increased (>10 kPa to 30 kPa), the composite film is further strained (compressive strain change from 31% to 57%, with 20 kPa change in pressure), but with a lower rate of change in the strain compared to Region Ⅰ, as shown in Figure 8b, and the rGO chain in Figure 10a starts to be squeezed together. Thus, the rGO structure collapsed, resulting in most of the 3D rGO-chains being squeezed together and touching each other, behaving like a quasi-bulk material, as illustrated in Figure 10b. In this situation, there are two competing mechanisms at the pressure of >10 kPa to 30 kPa, where the increase in the orthogonal strain increases the resistance as seen in Region I, but the 3D rGO chain structure collapsed when tightly squeezed and behaved as a quasi-bulk material. Hence, as the pressure increases, the resistance increases, as per that in Region I, but the creation of quasi-bulk materials reduces the resistance. Therefore, the overall increment in resistance in Region II increases at a slower rate due to these two competing mechanisms.

#### 2.3.3. Region III: Conduction Mechanism of the Sensor at High Pressure > 30 kPa

As can be seen in Figure 8b, the compressive strain change is only 17% when the pressure is increased from 30 kPa to 77 kPa. One reason could be that as the pressure increases beyond 30 kPa, the amount of compression is restricted by the sensor’s film thickness and further restricts the squeezing of the rGO structure. Therefore, almost all the 3D rGO structures collapse and behaves as a bulk material, causing the resistance to reduce slightly and then plateau off so no further collapse of the 3D rGO chain structure can occur (Figure 4b). 

## 3. Conclusions

Experiments were performed to study the conduction mechanisms of the piezoresistive rGO-based sensor from 0 kPa to 77 kPa. The increase in resistance to pressure exhibits three distinct regions. This could indicate the presence of two conduction mechanisms, which were investigated by measuring the variation in resistance with the temperature at high (28.5 kPa) and low (0 kPa) pressure. The variation in resistance with temperature and voltage suggests that at low pressure (0 kPa to 10 kPa), the conduction mechanism is dominated by Schottky/thermionic emission. At higher pressure (>10 kPa to 30 kPa), the Ohmic conduction mechanism becomes dominant. This can be explained by the deformation of the sensor under compressive load, with low pressures primarily causing a significant orthogonal tensile strain and the corresponding increase in barrier height. Therefore, resistance increases more rapidly with pressure, according to Schottky/thermionic emission. At the higher-pressure region (>10 kPa to 30 kPa), the 3D rGO chain structure begins to collapse, behaving similarly to quasi-bulk materials, resulting in a decrease in resistance. The onset of this conduction mechanism is overlaid onto the conduction due to Schottky emission, leading to the shallower gradient of resistance. Therefore, as the pressure further increases (>30 kPa), almost all the 3D rGO chain structures collapse and behave as a bulk material, causing the resistance to reduce slightly and then plateau off. Regions Ⅰ and II can be used as pressure sensors. Region I applies for low pressure with high sensitivity, while Region II is for medium pressure at the expense of a reduction in sensitivity. Region III is unsuitable and, therefore, cannot be applied to applications with high pressure, as the resistance reverses in direction in Region III. In conclusion, the flexible piezoresistive pressure sensor with outstanding sensitivity is a promising candidate to mimic the pressure-sensing performance of human skin where sensing ranges from 0 to 10 kPa are required. The sensor could also be applied in other applications such as object manipulations where pressures greater than 10 kPa are generated but limited to 30 kPa.

## Figures and Tables

**Table 2:** R^2^ values when curve-fitted with other conducting mechanisms.

Electrode-Limited Conduction Mechanism	Fowler–Nordheim (F–N) Tunneling	Direct Tunneling	Thermionic Field Emission	Schottky/thermionic emission
*I–V* plot	0.84	N.A.	0.80	0.97
*R–T* plot	N.A.	N.A.	0.85	0.95
Bulk-limited conduction mechanism	Poole–Frenkel (P–F) emission	Hopping conduction	Ohmic conduction	Ionic conduction
*I–V* plot	0.99	0.88	1	0.88
*R–T* plot	0.77	0.77	0.77	0.77

## Data Availability

Not applicable.
